# Nonalcoholic Fatty Liver Disease: Pathogenesis and Treatment in Traditional Chinese Medicine and Western Medicine

**DOI:** 10.1155/2020/8749564

**Published:** 2020-01-04

**Authors:** Tingting Shi, Li Wu, Wenjun Ma, Liping Ju, Minghui Bai, Xiaowei Chen, Shourong Liu, Xingxin Yang, Junping Shi

**Affiliations:** ^1^The Hangzhou Xixi Hospital Affiliated to Zhejiang Chinese Medical University, Hangzhou 310023, Zhejiang, China; ^2^Center of Clinical Evaluation, The First Affiliated Hospital of Zhejiang Chinese Medical University, 54 Youdian Road, Hangzhou 310006, Zhejiang, China; ^3^College of Pharmaceutical Science, Yunnan University of Traditional Chinese Medicine, 1076 Yuhua Road, Kunming 650500, Yunnan, China; ^4^Department of Liver Diseases, The Affiliated Hospital of Hangzhou Normal University, Hangzhou, Zhejiang, China

## Abstract

Nonalcoholic Fatty Liver Disease **(**NAFLD) is one of the most important causes of liver disease worldwide and probably destined to become the leading cause of end-stage liver disease in the coming decades, affecting both adults and children. Faced with the severe challenges for the prevention and control of NAFLD, this article discusses the understanding and mechanism of NAFLD from Chinese and Western medicine. Moreover, the progress regarding its treatment in both Chinese and Western medicine is also summarized. Both Chinese medicine and Western medicine have their own characteristics and clinical efficacy advantages in treating diseases. The purpose of this article is to hope that Chinese and Western medicine have complementary advantages, complementing each other to improve clinical NAFLD therapy prevention and treatment methods to receive more and more attention throughout the global medical community.

## 1. Introduction

The nonalcoholic fatty liver disease (NAFLD) is a clinicopathological syndrome caused by genetic, environmental, and metabolic stress-related factors and clinically manifested as fat accumulation in hepatocytes. Specifically, this accumulation exceeds 5% of hepatic wet weight, or changes in the fatty content took place in over 1/3 of hepatocytes per unit area even though the overconsumption of alcohol can be expressly excluded. Pathologically, it may develop into nonalcoholic fatty liver, nonalcoholic steatohepatitis (NASH), fatty hepatic fibrosis, and cirrhosis with disease progression [1, 2].

In recent years, with the improvement of the living standards, lifestyle change, aging of the population, and rise in obesity, NAFLD incidence remarkably increased in China, making it an increasingly important chronic noninfectious disease in this country [3]. According to a sampling survey of the general population in large- and medium-sized cities, the prevalence of NAFLD in Chinese adults is about 15% (6.3%–27%). Developed regions and affluent classes see higher prevalence of developing NAFLD, making NAFLD a liver disease second only to viral hepatitis [3]. Moreover, it is a critical health problem faced by children and teenagers [4].

Although NAFLD has mild symptoms, its damage is great, and its pathogenesis is not fully understood [5]. Both Chinese and Western medicine have their own explanation [6]. As regards traditional Chinese medicine (TCM), its pathogenesis mainly includes stagnation of liver-qi and catharsis disorder, leading to hindrance of functional activities of qi, liver-qi perversion attacking the stomach, qi disease involving blood, and poor blood flow, which finally induce NAFLD [7]. Chinese and international scholars agree that NAFLD is caused by multiple factors including insulin resistance, lipid metabolism disorder, and oxidative stress in Western medicine [8].

NAFLD can be prevented by following a scientific and reasonable dietary regimen, correcting poor dietary habits, reducing alcohol intake or quitting drinking, doing a moderate amount of aerobic exercise, and receiving a periodic physical examination [9–11]. However, when NAFLD is combined with inflammation and liver fibrosis, the effect of the etiological treatment is not ideal. Even if you successfully lose weight and insist on abstinence, it is difficult to ensure complete recovery of patients with fatty liver. The ideal treatment strategy is an early intervention and individualized drug treatment.

NAFLD medical treatment includes both TCM and Western medicine remedies [12]. TCM compounds are characterized by the concept of holism and differentiation treatment. With a multi-ingredient and multipath pharmacological action, TCM compounds are compatible with the complex pathogenesis of NAFLD, which can be used to mitigate NAFLD and some other associated diseases [13]. Western medicine currently used to treat NAFLD includes insulin resistance improvement and use of lipid-lowering drugs, antioxidants, and hepatocyte-protective agents, all promoting liver lipid metabolism and accelerating intrahepatic fat transport. Therefore, this article reviews the pathogenesis of NAFLD and the treatment progresses after using Chinese and Western medicine, and the possibilities of resolving NAFLD.

## 2. Knowledge and Mechanism of NAFLD in TCM and Western Medicine

### 2.1. Knowledge and Mechanism of NAFLD in TCM

No records are available regarding NAFLD in the TCM, but according to the symptoms and signs, it may be associated to what it is described as “lump at the left hypochondrium, y,” “accumulation, c,” “phlegmatic mass, h,” “hypochondriac pain, y,” “deep-rooted alcohol ulcer” and “damp obstruction” in TCM [14]. NAFLD is induced due to 3 reasons: improper diet, emotional disorder, and maladjustment in work and rest [15]. It is located mainly in the liver, closely related to lienal and renal functions. Its pathogenesis mainly includes stagnation of liver-qi and catharsis disorder, leading to hindrance of qi functional activities, liver-qi perversion attacking the stomach, qi disease involving blood, and poor blood flow, which finally induce NAFLD. Some hypochondriac pains are induced by phlegm evil caused by excessive rest after overconsumption of greasy and surfeit flavor in the course of the disease. Chronic hypochondriac pain induces liver, spleen and kidney dysfunction. If phlegm evil cannot be promptly removed, it continues to stay in the body and cause blood stasis, which finally develops into NAFLD [16]. TCM's holistic concept and differentiation treatment of NAFLD show their advantages and characteristics in the treatment of this complex metabolic disease. Moreover, the unique regimen and dietary therapy of TCM, as well as the emphasis on the importance of “preventive treatment of diseases, r,” play a more important role in future clinical prevention and treatment.

### 2.2. Knowledge and Mechanism of NAFLD in Western Medicine

In the history of the Western medicine, the knowledge of NAFLD was built through a long process of pathological events, imaging results, clinical syndrome, and independent disease [17]. Like alcoholic liver disease, NAFLD can lead to liver disability and death. Moreover, it is closely related to type-2 diabetes mellitus (T2DM) and arteriosclerotic, cardiovascular, cerebrovascular, and renal vascular diseases and extrahepatic malignant carcinoma [18]. Therefore, NAFLD is not merely hazardous to the liver; neither the diagnosis nor the treatment of NAFLD is merely restricted to alcoholic liver diseases. Actually, NAFLD has become a new challenge to the contemporary medicine [19]. In terms of threats to human health and social development, it is not second to alcoholic liver diseases and viral hepatitis.

NAFLD is a disease related to gene, environment, and metabolic stress [20]. Its causes include metabolic factors (e.g., diabetes mellitus), nutritional factors (e.g., obesity), drug, physical and chemical factors (e.g., corticosteroid), hereditary factors, systemic diseases, biological factors (e.g., microbial infection caused by virus and bacteria), mental, psychological and social factors, sedentary lifestyle, a high-fat and high-calories diet, and lack of exercise. In addition, NAFLD incidence remarkably increased with the increase of chemical pollution [21–23]. The occurrence of NAFLD can be attributed to a disease, or to several diseases that attack simultaneously or in sequence.

### 2.3. NAFLD Pathogenesis

Although many studies on NAFLD are available, its etiology and pathogenesis are still unclear. Chinese and international scholars agree that NAFLD is caused by multiple factors including:

1. *Insulin Resistance*. This term means that the sensitivity of the body to the produced insulin is reduced due to various causes. To maintain a stable blood sugar level, the body secretes even more insulin to compensate, resulting in hyperinsulinemia [24] leading to an increase in glycolysis to produce more fatty acids. Insulin resistance leads to an increase in free fatty acids due to its impact on lipid metabolism, which is beyond hepatic metabolic ability, resulting in excessive fatty acids accumulated in the liver. In addition, the secretion of very-low-density lipoprotein decreases so that TG increases.
2. *Lipid Metabolism Disorder*. The synthesis and secretion of triglyceride transfer protein and apolipoprotein decrease due to various causes. Triglycerides transferred by the liver are reduced; thus, they accumulate in the liver, resulting in NAFLD [25].
3. *Mitochondrial Dysfunction*. Mitochondria play a central role in complex processes, including the generation of energy and reactive oxygen species (ROS), maintaining calcium homeostasis, and adjusting apoptosis and lipid metabolism [26]. With the increasing number of studies, it has been demonstrated that mitochondrial dysfunction is an important mechanism of NAFLD and its aggravation, in which the following mechanisms may play a role [27–34]: (1) mitochondrial DNA damage; (2) disorders of energy metabolism; (3) oxidative stress and lipid peroxidation; (4) mitochondria-mediated hepatocellular apoptosis; (5) disorders of fatty acid metabolism; and (6) abnormal mitophagy. Thus, strategies to prevent mitochondrial damage or to manipulate mitochondrial function in a clinically useful manner may provide effective therapies to treat NAFLD.
4. *Oxidative Stress*. Oxidative stress occurs due to various causes. When a large amount of fat is stored in the liver, oxidative stress is significantly increased and energy is provided by excessive fatty acids. Long-term oxidative stress stimulates the liver, which may lead to nonalcoholic steatohepatitis (NASH) and cirrhosis [35]. Oxidative stress is considered as the most important mechanism causing damage to the liver in the “two-hit” hypothesis. The produced reactive oxygen species (ROS) increase damage to oxidative phosphorylation uncoupling and cell membrane leading to a vicious cycle that aggravates liver inflammation.

Oxidative stress mechanism in promoting the occurrence and progress of fatty liver disease induced by high-fat diet is as follows: (1) ROS formed by oxidative stress can combine with biological membrane phospholipids, enzymes, membrane receptor polyunsaturated fatty acid side chains, and macromolecules like nucleic acid, resulting in lipid peroxidation. Resulted lipid peroxides can increase endogenous ROS and toxicity and inhibit the antioxidant system. Lipid peroxidation can promote the synthesis and release of malondialdehyde (MDA) and B-hydroxylated nonene. Oxidative stress, lipid peroxidation, and mitochondrial damage further increase ROS/RNS production and accelerate and worsen fatty degeneration resulting in NASH and fibrosis [36]. (2) Lipids accumulate in liver cells. TNF-*α* is induced because oxidative stress and lipid peroxidation stimulate IKK*β*/NF-*κ*B inflammatory pathway. Then, the inflammatory factors IL-1 and IL-6 initiate and promote inflammation [37] and result in positive feedback to increase IKK*β* activity, forming a vicious IKK*β*/NF-*κ*B cycle. Such waterfall effect increases insulin signal transduction disorders and ultimately leads to intrahepatic insulin resistance and abnormal expression of PKB [38]. (3) As a molecule in the insulin signal pathway, PKB is very sensitive to oxygen-free radicals and its activity can be regulated by ROS. Free radicals can destroy its phosphorylation sites and affect its activity [39]. A study on co-antioxidant containing GSH on oxidative stress in T2DM rats based on microsomal lipid peroxidation model showed that the expression of P-PKB/PKB in liver and muscle tissues of these rats treated with antioxidant was significantly higher than that of normal control group [39]. Therefore, oxidative stress may be one of the initial factors that cause abnormal expression of PKB in the liver.

## 3. Breakthroughs in Medical Prevention and Treatment of NAFLD

NAFLD can be prevented by following a scientific and reasonable dietary regimen, correcting poor dietary habits, reducing alcohol intake or quitting drinking, doing a moderate amount of aerobic exercise, using various Chinese and Western drugs with care, and receiving a periodic physical examination [40, 41]. NAFLD is a disease, not a subhealth status. NAFLD treatment is a long-term comprehensive procedure, and the specific measures are as follows [5, 42]: (i) adopting a healthy lifestyle, including balanced diet and maintaining a positive attitude; (ii) reducing causes and inducements, including weight control and waistline reduction; (iii) preventing and treating metabolic disorders, such as taking antiplatelet, lipid-regulating, hypoglycemic, and antihypertensive drugs rationally in accordance with healthy blood viscosity, blood sugar, blood fat, blood pressure, and insulin resistance; (iv) using anti-inflammatory and liver-protecting drugs to treat NASH and progressive hepatic fibrosis, to reduce liver disability and mortality. Moreover, anti-inflammatory and liver-protecting drugs are both classified as TCM and Western medicine.

### 3.1. Breakthroughs in Prevention and Treatment with TCM

NAFLD, often associated with hyperlipidemia, hypertension, and diabetes mellitus, is a complex and holistic metabolic disease [43]. TCM compounds are characterized by the concept of holism and differentiation treatment. With a multi-ingredient and multipath pharmacological action, those TCM compounds are compatible with the complex pathogenesis of NAFLD, which can be used to mitigate NAFLD and some other associated diseases [44]. In Chinese clinical practice, TCM has unique advantages in the clinical treatment of NAFLD, and most patients with NAFLD received TCM treatment.

TCM stresses the differentiation treatment [8]. In other words, there are different types of syndromes in different patients with NAFLD, thus, different prescriptions need to be adopted for each specific treatment. The selection of prescriptions depends on the four properties of TCM (cold, hot, warm, cool), five flavors (sour, bitter, sweet, spicy, salty), and efficiency. According to clinical experience, NAFLD can be divided into the following types [45]: spleen-deficiency and phlegm-turbid stagnation (prescription: modified shenlingbaizhu powder), stagnation of liver-qi (prescription: modified Chaihu Shugan powder), accumulated damp-heat (prescription: modified gentianae decoction for purging liverfire), stasis blocking channels (prescription: modified decoction for promoting blood circulation), and efficiency of liver and kidney (prescription: modified Yiguan decoction with Liuwei Dihuang pills). The selection of clinical prescriptions should rely on the main symptom declining the other symptoms taken into consideration. There should be a little difference in the selected prescriptions, and a “differentiation treatment” might as well be adopted. TCM with a clear pharmacological action on fatty liver treatment can be selected according to the circumstances.

As regards the differentiation treatment, NAFLD is treated from a holistic perspective by ascertaining the cause before the determination of the treatment in a multiconditioning way to soothe liver-qi stagnation [46]. Basic therapies include spleen tonification and qi regulation, phlegm elimination and dehumidification, blood activation and phlegm emission, liver clearance, and promotion of bile secretion. Meanwhile, kidneys should be taken into consideration because they contain renal yin and renal yang [47]. In addition, they are the source of phlegm; thus, insufficiency of kidney qi and deficiency of kidney yang lead to qi dysfunction in transformation, thus aggravating phlegm-damp and stasis. Therefore, nephron warming can calm the liver and strengthen the spleen to further improve the therapeutic effect. The basic methods for NAFLD differentiation treatment include: lipid-regulating and liver-strengthening decoction, herba artemisiae scopariae Yueju decoction, dachaihu decoction, modified sini powder, qili preparation, lipid-lowering decoction, liver-soothing, spleen-strengthening and blood-quickening decoction, turbid-eliminating and liver-clearing decoction, fat-eliminating and liver-protecting decoction, Shennong liver lipid-eliminating pills, yang-warming and turbid-eliminating decoction, Wuchong prescription, Tiluo powder, Ganzhikang capsule, dampness-resolving and fat-eliminating decoction, liver-controlling-dredging and lipid-lowering decoction, fat-reducing decoction, lipid-regulating capsule, liver-soothing, circulation-promoting and stasis-removing decoction, and lipid-lowering and liver-benefiting pills [48–53] (Table 1). Furthermore, according to the literature and clinical experience, the Chinese medicinal herbs commonly used for treatment of NAFLD in clinical practice can be divided into the following types (Table 2).

**Figure 1:**
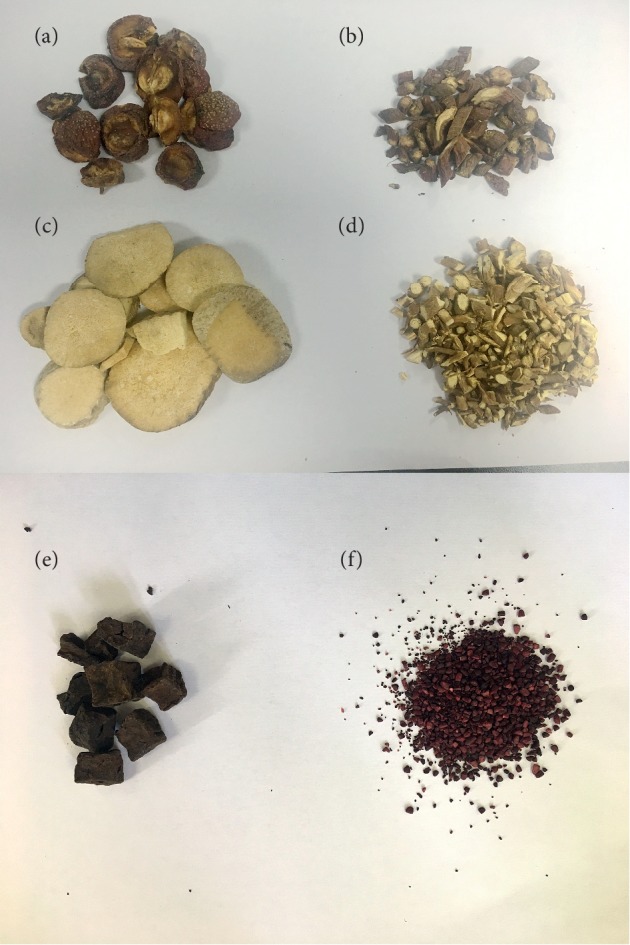
Six Chinese traditional medicine herbs. (a) Hawthorn. (b) Salvia miltiorrhiza. (c) Rhizoma alismatis. (d) Radix bupleuri. (e) Polygonum multiflorum. (f) Red yeast rice.

Some of the literature contains a summary of reports on fatty liver in main Chinese TCM journals in the past ten years and statistics of the defined daily dose system (DDDs) of TCM. The following medicinal herbs are the first six in order of frequency of use: Hawthorn, Salvia miltiorrhiza, Rhizoma alismatis, Radix bupleuri, Polygonum multiflorum, and Red yeast rice (Figure 1).

 
*Hawthorn*. Hawthorn is a classic TCM herb that can promote digestion and remove food retention. It has been widely studied in modern pharmacology. Ye et al. [63] found that hawthorn leaves flavonoids can significantly lower blood sugar and lipid and prevent fat accumulation in liver tissues to some extent. Yan et al. [64] found that hawthorn lowers lipid by affecting the PPAR*γ*-PPRE signal regulation system.
 
*Salvia Miltiorrhiza*. Salvia miltiorrhiza, bitter, a bit cold, acting on heart and liver channels, is effective in removing blood stasis, promoting fresh blood production, invigorating blood circulation and regulating the menstrual function. It can be used to improve the microcirculation and increase the hepatic blood flow. Salvia miltiorrhiza decoction has an antilipemic and anti-triglyceride effect on experimental animals such as rats and rabbits. The mechanism may be due to the promotion of fat oxidation in the liver and therefore lowers fat content in liver tissues. In addition, salvia miltiorrhiza and its active ingredients can scavenge free radicals and prevent lipid peroxidation. Guo et al. [65] proved that salvia miltiorrhiza could regulate the gastrointestinal function, alleviate hepatic steatosis and fibrosis, and activate receptor expression by enhancing peroxisome proliferators to effectively reverse high-fat-diet-induced hepatic steatosis and inflammatory changes. Modern research [66] shows that tanshinones and total phenolic acids are active constituents in salvia miltiorrhiza that can treat NAFLD. Specifically, NAFLD can be treated by enhancing lipid metabolism and antilipid peroxidation. In terms of the whole medical effect, total salvianolic acids are better than total tanshinones. Modern research shows that salvia miltiorrhiza promotes hepatocyte regeneration, thus repairing defective cells and inhibiting hepatic fibrosis. Now, it has been widely used in the treatment of fatty liver, hepatitis, and cirrhosis [67].
 
*Rhizoma Alismatis*. Rhizoma alismatis can inhibit the intake of exogenous triglyceride and cholesterol, affect endogenous cholesterol metabolism and inhibit intrahepatic triglyceride synthesis, thus improving liver fat metabolism. The extract of active ingredients of rhizoma alismatis possesses varying degrees of inhibitory actions on the fatty liver induced by low-protein diet and ethionine and a protective action on acute hepatic injuries caused by carbon tetrachloride because it can reduce intrahepatic fat mass and improve liver function.
 
*Radix Bupleuri*. Radix bupleuri, bitter, spicy, acting on liver and gallbladder channels can outthrust the exterior and abate fever, regulate qi activity, dredge the stagnation of liver-qi, and enhance yang. It is recorded in Herbal Classic as follows: “Radix bupleuri can remove the stagnation of qi in the heart, abdomen, intestines, and stomach; treat dyspepsia, chills, and fever; and promote metabolism.” Modern pharmacological studies demonstrate that radix bupleuri contains a substance called saikoside that can be used to improve capillary permeability, inhibit the release of inflammatory mediators, migration, and connection of white blood cells and the proliferation of tissue homogenates, thus lowering the level of serum ALT and AST in patients; protect the liver; increase superoxide (SOD) activity of hepatocytes; inhibit the synthesis of free radicals; and enhance oxidation resistance. This is the way it prevents and treats alcoholic liver disease [68–73]. In addition, saikoside can trigger a condensation of peritoneal macrophages to activate their expansibility and phagocytosis, kill intracellular saccharomycetes, improve acid phosphatase activity, and stimulate T-lymphoid cells and B-lymphocytes to participate in body immunoregulation [72]. Guo et al. [65] found that radix bupleuri combined with salvia miltiorrhiza could activate the expression of receptor *γ* by enhancing NF peroxisome proliferators, to inhibit lipid peroxidation and inflammatory mediator release in the hepatic tissues of rats with NAFLD, to effectively heal high-fat-diet-induced liver injuries.
 
*Polygonum Multiflorum*. Polygonum multiflorum is the tuberous root of the herbaceous Polygonum multiflorum, a Polygonaceae plant. Containing chrysophanol, emodin, lecithin, etc., the Polygonum multiflorum can stop cholesterol from depositing in the liver, reduce ALT and AST, and promote gastrointestinal peristalsis, thus lowering blood cholesterol, protecting the liver, and promoting metabolism. It is thereby used for the treatment of the fatty liver disease. Li et al.'s research shows that both water extracts (raw Polygonum multiflorum and radix polygoni multiflori preparata) and essential component (stilbene glycoside) of the Polygonum multiflorum can effectively alleviate NAFLD [73]; Lin et al.'s research shows that the essential components (free anthraquinones, combined anthraquinones and stilbenes) of the Polygonum multiflorum can relieve lipid abnormalities in adipohepatic liver cells L-02 [74]. Besides, Wang et al. have found that the water extracts (raw Polygonum multiflorum and radix polygoni multiflori preparata) of the Polygonum multiflorum can intervene in high-fat-induced NAFLD rats by regulating mitochondria membrane permeability [75].
 
*Red Yeast Rice*. Red yeast rice, warm-natured and sweetish, is effective in improving blood circulation and disperses stasis and invigorates spleen to promote digestion. Recent studies suggest that, containing multiple bioactive substances, such as statins, alkaloids, and flavones, the red yeast rice has extensive pharmacological effects including hypolipidemic, hypoglycemic, antihypertensive, and anti-inflammatory actions. Lee et al. found that red yeast rice has therapeutic potential in treating obesity and hyperlipidemia [76]. Fujimoto et al. found that red yeast rice is effective against obesity-related inflammation, insulin resistance, and NAFLD in metabolic syndrome-NAFLD mouse model irrespective of monacolin K levels. GABA and various peptides produced during fermentation were determined as the active constituents of red yeast rice [77]. Zhou et al. found that red yeast rice has the potential to ameliorate lipid metabolic disorders and therefore could be used as potential functional food ingredients for the prevention or treatment of hyperlipidemia and gut microbiota dysbiosis [78]. Patel found that red yeast rice has been validated to lower cholesterol; control high blood pressure; and confer antiflammation, hypoglycaemic, anticancer, and osteogenic properties [79]. Cicero et al. found that is the most effective cholesterol-lowering nutraceutical on the market. In particular, its effectiveness is directly related to the amount of monacolin K within the extract [80].

In conclusion, currently, many clinical workers combined disease differentiation with syndrome differentiation and used proper prescriptions to effectively improve liver function and clinical symptoms in patients with NAFLD, achieving a satisfactory clinical effect. Besides, Chinese medical scholars made some breakthroughs in pharmacological experiment studies on prevention and treatment of NAFLD. However, despite the considerable potential of TCM in the prevention and treatment of NAFLD, there are also some limits. Therefore, it is still necessary to further verify TCM mechanism of action on NAFLD to develop an easy-to-take effective clinical drug in the future.

### 3.2. Breakthroughs in Prevention and Treatment with Western Medicine

At present, treatment with NAFLD drugs mainly includes methods for improving insulin resistance, blood lipid regulation, antioxidants, hepatocyte protection, and microecological preparations, adipocytokines, and angiotensin converting enzyme inhibitors. The following four types are commonly used:

 
*Insulin Sensitizer*. Impaired glucose tolerance, fasting blood glucose rise, and abdominal obesity in T2DM patients can be controlled using metformin and thiazolidinedione compounds to improve insulin resistance and control blood sugar.
 
*Hypolipidemics*. If mixed hyperlipidemia or hyperlipidemia with at least 2 risk factors still persists 3–6 months after treatment of dyslipidemia with a basic therapy and/or slimming and hypoglycemic drugs, hypolipidemics such as fibrates statins or probucol can be used as adjuvant drugs.
 
*Drugs for Liver Diseases*. If NAFLD with hepatic dysfunctions and metabolic syndromes is still unhealed 3–6 months after treatment with a basic therapy, and the viable tissues in liver are diagnosed with a chronically progressive NASH, drugs for liver diseases can be used as an adjuvant treatment to achieve antioxidant, anti-inflammatory, and antifibrosis effects. A representative drug is polyene phosphatidylcholine (PPC). PPC is a drug for liver cell membrane repair. This type of drug can be combined with the liver cell membrane and cell organelle to enhance the integrity, stability, and fluidity of the membrane to restore the impaired liver function and enzymatic activity and regulate the hepatic energy metabolism to promote hepatocyte regeneration.
 
*Antioxidants*. Antioxidants have multiple mechanisms of action, such as antilipid peroxidation, antimitochondrial damage, auxoaction for hepatocyte protein synthesis, and antihepatocyte apoptosis. Antioxidants can also enhance the resistance of liver cell membrane against many injury factors. Representative antioxidants include silymarin, vitamin E, and NAC.

**Figure 2:**
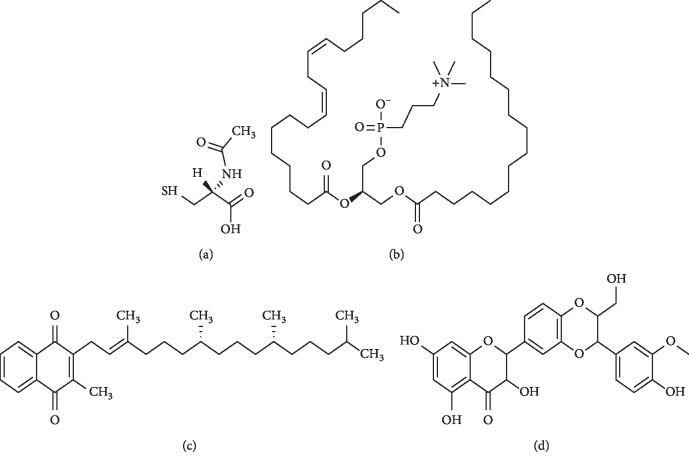
Chemical structure of antioxidant compounds discussed in the text. (a) N-acetylcysteine. (b) Polyene phosphatidylcholine. (c) Vitamin E. (d) Silymarin.

**Figure 3:**
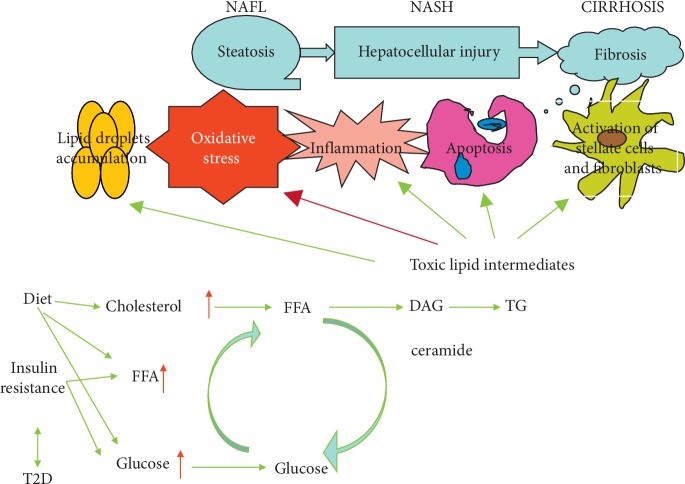
Scheme of the general mechanism of antioxidants on NAFLD. NAFL: nonalcoholic fatty liver; NASH: nonalcoholic steatohepatitis; T2D: type 2 diabetes; FFA: free fatty acids; DAG: diacylglycerol; TG: triglycerides; ox stress: oxidative stress.

Acetylcysteine, polyene phosphatidylcholine, vitamin E, and silymarin can be rationally selected and used according to drug performance, disease activity, and disease duration, but multiple drugs should not be used together (Figures 2 and 3).

#### 3.2.1. N-Acetylcysteine (NAC)

NAC is a compound containing an active mercapto group with strong anti-inflammatory and antioxidative effects. It can prevent free radical generation, remove generated free radicals and regulate cell metabolic activity. As a small molecule, NAC enters easily the cells. After entering the cells, it deacetylates and becomes a precursor synthesized by reduced glutathione to promote glutathione (GSH) synthesis, improve GSH content in tissues, and enhance the ability of tissues to resist oxidation and damage from free radicals [81–88]. Currently, NAC is also used in clinical applications. It is a precursor of intracellular reduced GSH and a common antioxidant. As it can effectively dissolve mucus, it is often used as an expectorant in respiratory tract infection medication [87–90]. In addition, NAC can prevent methanol and lipopolysaccharide poisoning and liver injury and liver failure caused by many drugs such as excessive acetaminophen. NAC molecule contains an active mercapto group which has a significant antagonism on oxygen-free radicals (including superoxide anion, hydrogen peroxide, and hydroxyl radical) in the body, reduces injury induced by oxygen stress, protects liver cells, and improves liver pathology [90–94].

In humans or experimental animals, the antioxidant NAC inhibits tissue damage caused by oxidative stress due to its anti-inflammatory and antioxidant properties [93, 94]. NAC is an antioxidant with direct and indirect antioxidant action used in clinical practice [95]. NAC significantly alleviates remote liver injury in terms of morphology and transaminases, in a dose-depended manner, and reduces lipid peroxidation in serum.

NAC is a direct precursor of GSH synthesis. This drug is a nutritional supplement that replenishes intracellular GSH. Indeed, it is a safe antidote, at various doses, for GSH deficiency in a wide range of metabolic disorders, pulmonary diseases, neurotoxicity, hepatotoxicity, and immunotoxicity. NAC treatment attenuates arsenic-induced oxidative stress in the liver and thus prevents hepatocyte apoptosis [96–99]. Recently, NAC has been shown to exert a protective effect against insulin resistance and diabetes in rats [99–104]. Samuhasaneeto et al. [103] found that NAC can improve oxidative stress and liver morphology in rats with NASH induced by a high-fat diet. Thong-Ngam et al. and Shi et al. [104, 105] found that intragastric administration of NAC to high-fat-diet rats can improve oxidative stress and morphology changes in rat liver. This study also found that NAC intragastric administration with the diet control has a better effect in the improvement of oxidative stress and histopathology changes than the treatment by the independent diet control. NAC treatment by means of controlling diet and therapeutic dose can reduce lipid peroxidation and improve liver pathology. Zhang et al. [106] observed the effects of different drug interventions on insulin resistance induced by intravenous infusion of lipid emulsion in normal rats and found that the insulin resistance of rats treated with NAC can be significantly improved through the establishment of model with hyperinsulin-euglycemic clamp. Konarkowska and his colleagues [107] found that NAC can respond to ROS and regulate cellular redox reactions by increasing intracellular GSH levels and/or acting as a SH-group reductant to prevent amylin-induced apoptosis in human cells.

The current study suggests a decrease in the size of the spleen in NAFLD patients receiving NAC, which may reflect a reduction in fatty infiltration. These NAC effects can be evaluated in detail by reviewing liver specimens after the intervention, and may be improved after a longer duration [108]. Uzun et al. demonstrated that NAC reduces oxidative stress induced by NAFLD or resection [109]. The use of NAC as an antioxidant and anti-inflammatory agent appears to be a valuable adjuvant therapy for NAFLD, beyond its individual effects, as evidenced by biochemical and histological results [110]. Samuhasaneeto et al. [111] observed that rats in a high-fat-diet (HFD) model of NASH induction receiving NAC by oral administration have total GSH and hepatic MDA back to normal levels. In another animal model obtained by the induction of NASH by HFD via enteral nutrition, Baumgardner et al. demonstrated that NAC prevents many aspects of NASH progression by decreasing the development of oxidative stress (as a result of decreased MDA), but it is unable to block the development of steatosis [112].

#### 3.2.2. Polyene phosphatidylcholine (PPC)

The main ingredients of PPC capsules are several, including B vitamins and E vitamins. Polyenoic phospholipid is necessary for physiological processes of the body [113]. It can combine with liver cell and its organelle and become part of cell biological membrane. Moreover, it can repair damaged liver cell membrane biological structure to maintain the mobility and stability of liver cell membrane and recover damaged liver cells and transaminase. Furthermore, it can reduce oxidative stress and lipid peroxidation, inhibit hepatocyte apoptosis, reduce hepatic stellate cell activation after inflammatory reaction, significantly lower transaminase level, and effectively prevent liver cell degeneration and inflammatory fibrosis [114–118] to protect liver cells. In addition, it can affect lipid metabolism in the body and promote the formation of numerous cell soluble plasmids and rapid decomposition of fat in the body, ultimately inhibiting the accumulation of fat in the body.

PPC normalizes the metabolism of lipids and proteins and improves the detoxification function in the cell and restores the cell structure [117]. PPC also contains polyunsaturated choline that can significantly prevent liver cells from abnormal degeneration, inflammation, and fibrosis in addition to limiting the reduction in mitochondrial enzyme activity and increasing membrane protease bioactivity [118].

Administration of phosphatidylcholines with PPC results in the restoration of the level of phosphatidylcholines and break of the vicious cycle through its main and highly bioavailable component [119]. PPC is the main component of lecithin, and it is an integral part of cell membranes, essential for their structural and functional integrity. Cell membranes act like gatekeepers, allowing nutrients into the cells but blocking damaging toxins from gaining entrance. PPC enhances cell membrane function throughout the body [120]. Horejsová et al. [121] showed that daily PPC supplementation (along with low doses of fatty acids, B vitamins, and vitamin E) reduces fatty liver symptoms within 6 months in more than half of the study participants. Another study by Li et al. [122] indicated that PPC increases the breakdown of collagen, the connective-tissue protein that tends to accumulate in liver disease, promoting the scarring behind fibrosis and cirrhosis.

The main active ingredient of polyene phosphatidylcholine is 1,2-dilinoleoyl-phosphatidylcholine. After oral administration, it is mostly absorbed by the small intestine into blood and reaches and gathers in the liver first. Then, it enters liver cells and combines with liver cell membrane to protect and repair liver cells, promote their regeneration, reduce free radical attacks, recover and enhance the activity of lipid metabolism enzymes such as GSH reductase and catalase, improve lipid metabolism, and reduce lipid peroxidation damage. It can transform intrahepatic lipid components into those which are easily absorbed and metabolized and reduce blood triglyceride, cholesterol levels [123] and free radicals. In addition, it can enhance the activity of catalase, SOD and GSH reductase, reduce oxidative stress and lipid peroxidation, inhibit hepatocyte apoptosis, reduce inflammatory response, and inhibit hepatic stellate cell activation to protect liver cells from damage [115, 124], exerting its therapeutic effects.

PPC is extracted from soy and it is rich in polyunsaturated fatty acid, including linoleic acid, linolenic acid, and oleic acid [125]. Polyunsaturated fatty acid, also known as essential fatty acid, cannot be autonomously synthetized in the human body and must be supplied by food [126]. Therefore, PPC plays a role in numerous functions, such as anti-inflammation, antioxidation, and immune regulation function [127, 128]. In clinical practice, PPC is widely used in various types of liver disease treatment [129]. Cao et al. observed a PPC effect on NASH through regulating oxidative balance, inhibiting inflammatory factors, and NF-*κ*B signaling pathway [130]. Kim et al. observed that PPC specifically affects adipocytes and has less effect on preadipocyte viability. It can therefore be a promising agent to selectively reduce adipose tissue mass [131]. The treatment of NASH patients with PPC can improve their liver function and lower blood lipids [132, 133]. He et al. observed that PPC intervention may partially attenuate the inflammatory response by adjusting the imbalance of Th17/Treg cells, thus ameliorating the progression of NAFLD [134].

#### 3.2.3. Vitamin E

Vitamin E has eight isoforms that can be categorised into tocopherol isoforms, which have a saturated side chain on the chromanol ring, and into tocotrienol isoforms, which have an unsaturated side chain. All the isoforms of vitamin E have some antioxidant activity. RRR-*α*-tocopherol has the highest *in vivo* bioactivity among all vitamin E isomers. It binds a specific transport protein, the *α*-tocopherol transfer protein (*α*-TTP) [135]. Due to its structure and physical-chemical properties, vitamin E is one of the key antioxidants found in nature. After reaction with peroxyl radicals, an *α*-tocopheroxyl radical is formed.

Vitamin E is the key essential lipophilic (fat-soluble) antioxidant located in human cell membranes, protecting them from oxidative damage. The essential role of vitamin E in the human antioxidant defence system has been reevaluated by the European Food Safety Authority expert panel, which concluded that scientific evidence indicates that vitamin E contributes to the protection of cell constituents from oxidative damage [136]. *In vivo* and *in vitro* studies showed that vitamin E functions as a chain-breaking antioxidant acting to protect unsaturated lipids from peroxidation by scavenging peroxyl radicals [137].

In zebrafish, a model organism for lipid metabolism, vitamin E deficiency leads to a decrease in the content of highly unsaturated fatty acids [138], perhaps because of their consumption by peroxidative processes. The main function of vitamin E is to protect lipids from oxidative damage. Increased oxidative stress occurs in NAFLD. Among antioxidants, vitamin E has the most significant evidence supporting its use in NAFLD. In the PIVENS study [139], vitamin E supplementation, 800 UI/d, resulted in a significant improvement of NASH pathological features. Vitamin E regimen significantly decreases NAFLD liver fat score [140]. Vitamin E 400 UI/day is associated with a significant decrease in liver steatosis, ballooning degeneration of hepatocytes, and pericellular fibrosis in nondiabetic noncirrhotic patients with NASH [141].

Vitamin E showed some morphological improvements in NASH [142] and effectively improves gene expression associated with eukaryotic initiation factor-2 signaling, which is suppressed in NASH by endoplasmic reticulum stress in the liver [143]. Supplementation of more than 55.5 mg/kg vitamin E may improve growth and increase of *n* − 3 long chain polyunsaturated fatty acids content in blunt snout bream, while the expressions of PPAR-*α*, PPAR-*β*, and PPAR-*γ* in liver were downregulated, which is beneficial to human consumer [144]. Vitamin E affects the antioxidant parameters of the examined tissues [145]. Vitamin E supplementation is the effective treatment in lowering liver enzymes, leptin, FBS, insulin, TG, TC, and LDL-C among NAFLD patients [146]. In addition, it has had some efficacy in pediatric NASH [147].

#### 3.2.4. Silymarin

Silymarin is traditionally used as an adjuvant for treatment of liver diseases in Europe. In the late 1960s, Wagner (a West German pharmacist) and his colleagues first obtained the active ingredient of silybum marianum by extracting it from the seed capsule of the Compositae medical plant Silybum marianum. Silymarin contains several compounds including silybin, silydianin, silychristin, and isosilybin [148, 149]. Among all ingredients, silybinin has the highest content and activity [150, 151].

Silymarin is used in chronic hepatitis, cirrhosis, fatty liver, alcoholic liver, and toxic liver injury because it has antioxidant, anti-inflammatory/immunomodulatory, and antifibrotic properties in various *in vitro* and animal models [152] and may be useful in patients with NAFLD [153]. Due to its ability to scavenge free radicals, silymarin has antiperoxidation activity. Lipid peroxidation (which causes damage to cell membrane) can be blocked or prevented by silymarin. In addition, silymarin stimulates protein synthesis and normalizes phospholipid metabolism in damaged liver cells. In short, the greatest contribution of silymarin is stabilizing liver cell membrane [154]. It can also prevent or avoid the loss of soluble cellular components (such as transaminase) and prevent certain hepatotoxic substances (such as *α*-amanitin) from penetrating into cells. Silymarin stimulates the activity of RNA-polymerase in the nucleus to improve protein synthesis, thus the synthesis of ribosomal RNA in liver cells and synthesis of substantial structural and functional proteins (enzymes). Therefore, silymarin can enhance the repairing and regeneration of liver cells. It can prevent the occurrence or delay the process of NAFLD.

Indeed, it can significantly improve prothrombin time and liver function in NAFLD patients. Studies showed that it has many pharmacological properties, such as removing harmful oxygen-free radicals from the body, preventing cytotoxicity, increasing GSH levels in the body, reducing lipid peroxidation, protecting liver cell biological membrane from damage caused by harmful factors, promoting regeneration of liver cells, preventing liver fibrosis [155], regulating the body's immune system [156, 157], stimulating protein molecule synthesis, and protecting liver cell DNA synthesis by inhibiting NF-*κ*B [158].

Oxidative stress is thought to play a central role in the etiology of NASH, a specific subset of NAFLD and is hypothesized to represent a “second hit” triggering the necroinflammatory response characteristic of NASH [159]. Therefore, the antioxidant properties of silymarin may be particularly beneficial as a treatment for NASH because patients have significantly increased levels of serum lipid peroxidation products [160] and other oxidative stress markers and decreased levels of antioxidant enzymes [161]. Schrieber et al. showed that the disposition of silymarin, an herbal medicine widely used by patients with liver disease, is significantly altered in patients with liver disease [162]. Silymarin hepatic protection has been widely demonstrated in animal models and in clinical setting [163–168]. It is effective in the treatment of obesity-related NASH [163], prevents the establishment of alcoholic steatohepatitis [165] and silymarin pretreatment reduced the accumulation of fat in the liver of mice that received irinotecan. This supports the assumption that lipid accumulation occurs due to mitochondrial dysfunction [167]. Low doses of silymarin prevent the appearance of clinical signs highly correlated to NASH [168, 169] such as lobular inflammation, steatosis, and vacuolation and also prevent the expression of *α*-smooth muscle actin, a known fibrosis marker associated with advanced NASH. On the other hand, higher doses promote liver morphological damage, with increased levels of serum hepatic transaminases and higher expression of *α*-smooth muscle actin.

## 4. Conclusions and Summary

NAFLD, which affects approximately 25% of the global adults, is the most common chronic liver disease worldwide. Like adults, children and teenagers are also susceptible to NAFLD and NASH.

Along with the increasing prevalence of obesity and T2DM, which are closely related to NAFLD, the global morbidity of NAFLD and NASH has constantly risen, gradually increasing NASH-related clinical burden, financial burden, and life burden on patients. More severely, today there is still a lack of ideal methods for noninvasive diagnosis of NASH and effective drugs to combat NASH. If this situation carries on, NAFLD may cause a serious health crisis in the next several decades.

At present, we need to carry out international collaborative research in a number of areas to better understand the differences and similarities in NASH among different parts of the world, and develop a better noninvasive detection method to detect, and predict and monitor NASH and fibrosis. At present, a number of drugs for NASH therapy and antihepatic fibrosis are undergoing clinical trials, with their exact efficacy and safety to be determined. Due to the lack of effective drugs, currently the only thing to do is to change the lifestyle and effectively control body weight.

## Figures and Tables

**Table 1:** Basic methods used in NAFLD differentiation treatment.

Types	Chinese medicinal herbs	Effect
Chai Hu Li Zhong Tang [52]	Bupleurum, Scutellaria, ginger Pinellia, Codonopsis, Atractylodes, Poria, turmeric, Zhigancao, ginger, and jujube	↓TG, TC, LDL-C, AST, ALT, and insulin ↑HDL-C; activating AMPK*α*

Ganshutang decoction [53]	Coptidis chinensise, pinellia ternata, trichosanthes kirilowii, rhizoma curcumae longae concisa, and aralia taibaiensis	↓TG and LDL-C↑HDL-C and MDA Improving fatty degeneration and membrane fluidity of hepatocellular mitochondria. Protecting the structure and function of liver sieve and hindering lipid peroxides from being generated

Ruangan compound [54]	Selfheal, alga, desmodium, oyster and polygonum multiflorum	↓MDA; ↑SOD Correcting unbalanced oxidation and antioxidation; hindering hepatic stellate cells from being activated and their proliferation and prevent extracellular matrixes from anomalous deposition

Ganzheng compound [55]	Radix bupleuri, salvia miltiorrhiza, rhizoma alismatis, and pinellia ternata	↓TNF-*α*; ↑Adiponectin ↓CYP2E1 Reverse hepatic steatosis and inflammation

Aromatic fat-reducing drug [56]	Agastache stem, hypericum japonicum thunb, five leaf akebia fruit, serissa foetida comm., white peony root, herba artemisiae scopariae, angelica sinensis, and poria cocos	↓ALT, AST, TG and TC

Ahome-made spleen-invigorating and dampness-eliminating decoction [57]	Radix pseudostellariae, poria cocos, bighead atractylodes rhizome, radix glycyrrhizae, pinellia ternata, and dried tangerine or orange peel	↓TC, TG, ALT, AST and GGT A good liver-protecting and antilipemic effect

Fuzilizhong decoction [58]	Ginseng radix et rhizoma, Rhizoma atractylodis macrocephalae, Radix glycyrrhizae preparata, Zingiberisrhizoma, Aconiti Lateralis radix praeparata	Limiting pathological changes; lowering the content of blood lipid Improving liver function and decreasing liver index

A compound made from panax japonicus, salvia miltiorrhiza, and hawthorn [59]	Panax japonicus, salvia miltiorrhiza, and hawthorn	Antioxidant activity

A phlegm-dissolving and stasis-removing herb [60]	Processed rhizoma pinelliae, Fujian rhizoma alismatis, alga, salvia miltiorrhiza, curcuma wenyujin, stir-baked radix bupleuri with vinegar, radix et rhizoma rhei, semen cassiae, roasted bighead atractylodes rhizome, and raw astragalus	↓TC, TG, FFA, ALT, and AST; promoting the upregulated expression of LXR*α* mRNA and ABCA1 to limit liver pathological changes

TangGanJian [61]	White paeony root, angelica sinensis, Bupleurumchinense, wolfiporia cocos, atractylodes macrocephala koidz, artemisia capillaris thunb, polygonum cuspidatum sieb. et zucc, Schisandra chinensis (Turcz.) baill, and coptis chinensis franch	↓TNF-*α*, IL-6, IL-1*β*, CRP ↑GSH-PX and SOD; modulating the inflammatory response and the capacity of oxidative stress

Shugan-Jianpi Recipe [62]	Chai hu, chen pi, chuan xiong, Xiang fu, zhi qiao, Bai shao, and gan cao	↓IL-1*β*, IL-6, and TNF-*α* ↓p38 MAPK/NF-*κ*BmRNA and a protein

**Table 2:** Classification of Chinese medicinal herbs commonly used for NAFLD treatment in clinical practiceaccording to the literature and clinical experience.

Type	The Chinese medicinal herbs
Circulation-promoting and stasis-removing	Salvia miltiorrhiza, white peony root, hawthorn, lycopus lucidus, angelica sinensis, radix curcuma, cattail pollen, peach seed, prepared rhubarb, red peony root, polygonum cuspidate, and dried radix rehmanniae.

Heat-clearing and damp-drying	Scutellaria, cape jasmine, coptis chinensis, herba artemisiae scopariae, phellodendron, and polygonum cuspidate.

Spleen-invigorating and qi-supplementing	Bighead atractylodes rhizome, astragalus, dried tangerine or orange peel, and codonopsis pilosula

Dampness elimination with aromatics	Fresh lotus leaf, lotus petiole, rhizoma atractylodis, fructus amomi, round cardamom fruit, and flos magnoliae officinalis

Spleen-invigorating for dampness elimination	Coix seed, poria cocos, and bighead atractylodes rhizome
Diuresis-inducing with bland drug	Rhizoma alismatis and poria cocos

Spleen-invigorating for removing food retention	Hawthorn and medicated leaven

Liver-soothing for qi-regulating	Bupleurum, radix curcumae, fructus aurantii, and mint

Phlegm-reducing and masses-resolving	Zhejiang fritillary, alga, kelp, and phizome pinelliae

Liver blood-nourishing	Fructus lycii, angelica sinensis, white peony root, radix polygoni multiflori preparata, and fructus ligustri lucidi

Yin-nourishing and liquid-engendering	Radix glehniae, tuber of dwarf lilyturf, dried radix rehmanniae, polygonatum kingianum, radix polygonati officinalis, and radices trichosanthis

Liver and kidney-tonifying	Prepared rehmannia root, fructus corni, parasitic loranthus, radix polygoni multiflori preparata, fructus lycii, fructus schizandrae, and achyranthes bidentata

Heat-clearing for liver-calming	Chrysanthemum morifolium ramat, semen cassia, scutellaria, and frostbitten mulberry leaf

## Additional Points

*Core Tip.* NAFLD has emerged as the most common chronic liver disease worldwide. Faced with the severe challenges for the prevention and control of NAFLD, this article discusses the understanding and mechanism of NAFLD from Chinese and Western medicine. The purpose of this article is to hope that Chinese and Western medicine have complementary advantages, complementing each other to improve clinical NAFLD therapy and prevention and treatment methods to receive more and more attention throughout the global medical community.
